# Life at the crossroads: the nuclear LINC complex and vascular mechanotransduction

**DOI:** 10.3389/fphys.2024.1411995

**Published:** 2024-05-20

**Authors:** Pauline Bougaran, Victoria L. Bautch

**Affiliations:** ^1^ Department of Biology, The University of North Carolina, Chapel Hill, NC, United States; ^2^ McAllister Heart Institute, The University of North Carolina, Chapel Hill, NC, United States

**Keywords:** endothelial cell, nucleus, LINC complex, SUN protein, mechanotransduction, cytoskeleton, lamin, vascular disease

## Abstract

Vascular endothelial cells line the inner surface of all blood vessels, where they are exposed to polarized mechanical forces throughout their lifespan. Both basal substrate interactions and apical blood flow-induced shear stress regulate blood vessel development, remodeling, and maintenance of vascular homeostasis. Disruption of these interactions leads to dysfunction and vascular pathologies, although how forces are sensed and integrated to affect endothelial cell behaviors is incompletely understood. Recently the endothelial cell nucleus has emerged as a prominent force-transducing organelle that participates in vascular mechanotransduction, via communication to and from cell-cell and cell-matrix junctions. The LINC complex, composed of SUN and nesprin proteins, spans the nuclear membranes and connects the nuclear lamina, the nuclear envelope, and the cytoskeleton. Here we review LINC complex involvement in endothelial cell mechanotransduction, describe unique and overlapping functions of each LINC complex component, and consider emerging evidence that two major SUN proteins, SUN1 and SUN2, orchestrate a complex interplay that extends outward to cell-cell and cell-matrix junctions and inward to interactions within the nucleus and chromatin. We discuss these findings in relation to vascular pathologies such as Hutchinson-Gilford progeria syndrome, a premature aging disorder with cardiovascular impairment. More knowledge of LINC complex regulation and function will help to understand how the nucleus participates in endothelial cell force sensing and how dysfunction leads to cardiovascular disease.

## 1 Introduction

Unlike many other tissues, blood vessels are exposed to mechanical forces that strongly influence both vessel development and vascular function (Roux et al., 2020), and inappropriate or incomplete responses to these forces trigger vascular disease (Marchuk et al., 2003; Baeyens et al., 2016; Humphrey and Schwartz, 2021; Tabosh et al., 2024). Early work showed that the endothelial cells lining all blood vessels align their cytoskeleton and nucleus-centrosome-Golgi axis to the shear stress vector imparted by laminar blood flow (Reidy and Langille, 1980; Dewey et al., 1981; Remuzzi et al., 1984). Other forces created by blood flow, including cyclic strain and outward pressure, also influence endothelial cell responses and those of support cells such as vascular smooth muscle (Hoefer et al., 2013; Campinho et al., 2020). Vascular flow patterns strongly coincide with disease development *in vivo*; for example, areas of disturbed flow such as vessel branch points are prone to atherosclerotic plaque development. Plaque formation leads to reduced blood flow, additional turbulence, and in some cases, plaque rupture and the formation of blood clots.

Mechanical forces generated by laminar blood flow are crucial for vascular remodeling once a primitive vascular plexus forms during development. Vascular remodeling leads to large arterial conduits that branch into smaller arterioles, then into small diameter capillaries that eventually lead to larger caliber venules and veins that return blood to the heart on the venous side. This pattern results from the regression of early vessel segments that experience reduced flow, while segments with elevated blood flow remain and enlarge (Potente et al., 2011). Endothelial cell responses to blood flow strongly contribute to the hierarchical distribution of vessel diameters in most vascular beds, and several lines of evidence support the model that endothelial cells migrate from areas of low flow to areas of higher flow as the vascular network is remodeled and patterned (Udan et al., 2013; Xu et al., 2014; Franco et al., 2015; Pitulescu et al., 2017; Lee et al., 2022). Although initial arterial-venous identity is not flow-dependent, the maintenance of arterial-venous status relies on shear stress, and higher magnitude shear vectors maintain arterial identity while lower vectors maintain venous identity (le Noble et al., 2004; Chong et al., 2011). Finally, laminar flow is critical to maintain vascular homeostasis and an anti-inflammatory state in endothelial cells. Disturbed flow, or the inability of endothelial cells to respond to laminar flow, sets up a pro-inflammatory endothelial cell phenotype that often leads to disease. Thus, blood vessel formation and function are intimately linked to proper endothelial cell responses to mechanical signals.

Endothelial cells form a flattened monolayer on the inner vessel wall that is a single cell layer thick, with very little cytoplasm between the apical (luminal) and basal (abluminal) endothelial surfaces. In this topology, the endothelial cell nucleus extends above the apical plane of the cytoplasm, essentially leaving it exposed to shear forces created by blood flow. Despite this topology and evidence that the nucleus is required for flow-mediated endothelial cell functions and migration (Tkachenko et al., 2013; Guilluy et al., 2014; Graham et al., 2018), how the nucleus acts as a mechanotransducer in endothelial cells is poorly understood, and how the nucleus integrates cytoplasmic mechanotransduction inputs has not been extensively investigated. However, nuclear integrity in vascular cells is important for proper vessel function and maintenance, and human mutations in nuclear-localized proteins such as lamin-A lead to diseases with cardiovascular dysfunction such as Hutchinson-Gilford progeria Syndrome (Östlund et al., 2019).

Several molecules and molecular complexes that reside in the endothelial cell plasma membrane, often at cell-cell junctions, transduce flow-generated mechanical forces on the apical side. These structures, such as PECAM1/VE-cadherin/VEGFR2, Piezo channels, and plexin D1, translate mechanical inputs into cellular signaling readouts (Tzima et al., 2005; Duchemin et al., 2019; Mehta et al., 2020), that in turn regulate flow-responsive signaling pathways in endothelial cells. The BMP pathway is one example of a flow-responsive signaling axis, where key signaling components likely do not act as direct sensors but are strongly regulated by flow-mediated inputs (Han et al., 2021; Desroches-Castan et al., 2022; Kulikauskas et al., 2022). In contrast to the extensive literature on endothelial cell signaling in general, how the nucleus participates in mechanotransduction is poorly understood. The LINC (Linker of Nucleoskeleton and Cytoskeleton) complex resides in the nuclear envelope and bridges the nucleus and cytoskeleton, and it has recently been implicated in mechanotransduction processes (Janota et al., 2020; King, 2023). Although most of the published work is in non-vascular cells, some recent studies examine LINC complex functions in the vasculature (Mannion and Holmgren, 2023).

**FIGURE 1:**
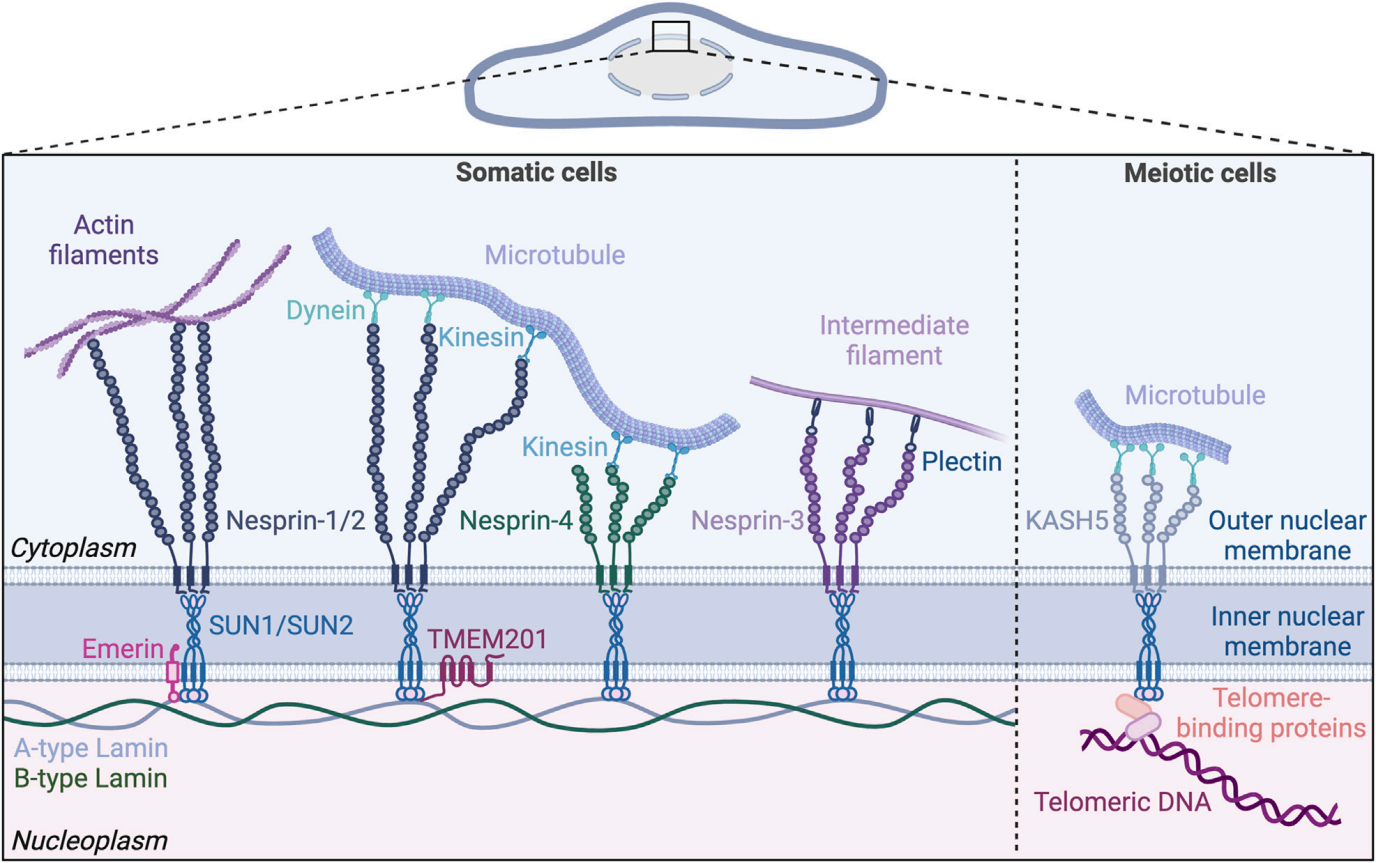
The nuclear LINC (Linker of Nucleoskeleton and Cytoskeleton) complex. The LINC complex resides in the nuclear envelope and bridges the inner and outer nuclear membranes. Inner nuclear membrane proteins SUN1 and SUN2 bind both A-type and B-type lamins in the nucleoplasm. On the cytoplasmic side, SUN proteins form trimers and bind KASH proteins anchored in the outer nuclear membrane. KASH proteins (nesprins 1–4) extend into the cytoplasm and engage with the cytoskeleton. Nesprin-1 and 2 directly interact with actin filaments and, via motor proteins, with microtubules. Nesprin-4 also mediates microtubule binding by interacting with kinesin, while nesprin-3 indirectly binds to intermediate filaments via plectin. The lamin-binding proteins emerin and TMEM201 reside in the inner nuclear membrane and bind to the SUN proteins. In meiotic cells, the LINC complex formed by SUN/KASH-5/dynein is involved in chromosome movement by tethering the telomeres to microtubules. Created with BioRender.com.

Here we provide a brief overview of the LINC complex, followed by a specific focus on its function in vascular endothelial cells. Other recent excellent reviews discuss the role of the LINC complex in non-vascular cells and in non-endothelial vascular cells (Iyer et al., 2021; Salvador and Iruela-Arispe, 2022). The LINC complex consists of inner nuclear membrane-localized SUN (Sad1, UNC84) proteins that interact with lamin intermediate filaments in the nucleus and KASH (Klarsicht/ANC-1/Syne Homology) proteins in the outer nuclear membrane that in turn interact with the actin, microtubule, and intermediate filament cytoskeletons outside the nucleus (Starr, 2019; McGillivary et al., 2023). Given the promiscuous nature of KASH protein interactions with SUN proteins and cytoskeletal components, this review primarily focuses on the two major SUN proteins. We picture the SUN proteins sitting in the inner nuclear membrane of the endothelial cell lining the blood vessel **(** Figure 1). Like Janus, they face both inward to the nucleus proper and outward to the cytoplasm via KASH interactions, with outside-in interactions transmitting cytoskeletal changes to the nucleus and chromatin, while inside-out interactions integrate nuclear inputs for transmission to the cytoplasm and plasma membrane (Hieda, 2019). We describe cell-based and *in vivo* studies that inform our current knowledge of endothelial cell SUN protein function, we discuss links to cardiovascular pathologies, and we highlight future directions that will inform how the endothelial cell integrates nuclear mechanotransduction to respond to forces that regulate vascular function and disease.

## 2 The LINC complex: a bridge between the nucleus and cytoskeleton

The mechanosensing properties of the nucleus are the focus of recent work (Kirby and Lammerding, 2018; Östlund et al., 2019). The nucleoplasm contains the genetic information of the cell, DNA, organized as chromatin. The nucleoplasm is enclosed by the nuclear envelope, which is a double membrane consisting of inner and outer layers separated by perinuclear space. Nuclear pore complexes span both membranes and facilitate exchange between the nucleoplasm and cytoplasm (Gerace et al., 1978; Gerace and Burke, 1988). On the nucleus side, the inner membrane is next to an interconnected network of A-type (lamin-A and C) and B-type (lamin-B1 and B2) lamin intermediate filaments that form the nuclear lamina (Gerace et al., 1984).

**FIGURE 2:**
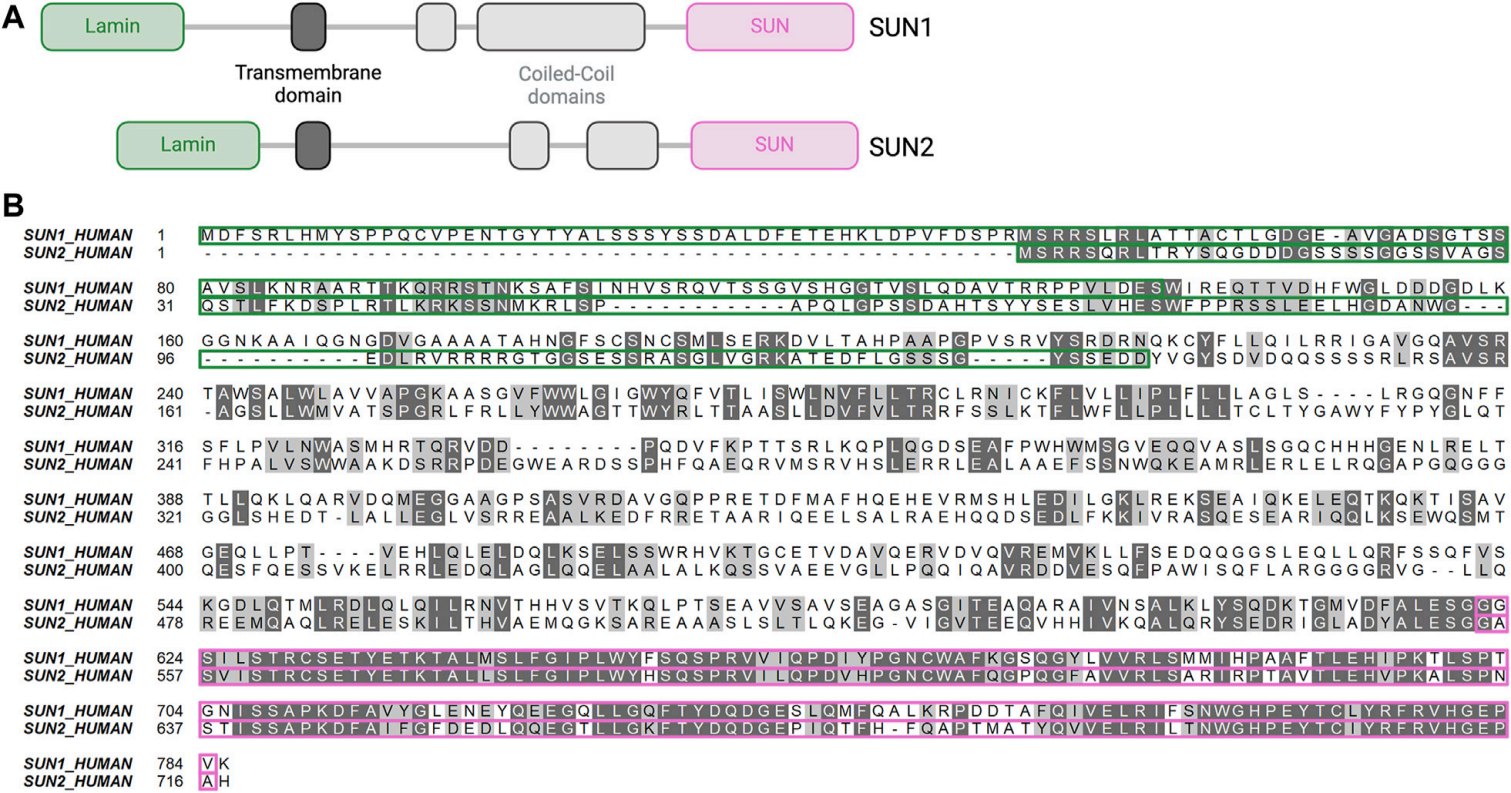
Structure and alignment of SUN1 and SUN2. **(A)** Primary structure of SUN1 and SUN2. Lamin-binding domain (green), transmembrane domain (dark grey), cytoplasmic domain with coiled-coil domains (light grey), SUN domain (pink). Lamin domains bind lamins, SUN domains bind KASH proteins. Created with BioRender.com. **(B)** Amino-acid sequence alignment of SUN1 and SUN2. Identical amino acids (dark grey) and similar amino acids (light grey) show high degree of sequence conservation of the SUN domain (pink highlight) compared to the lamin-binding domain (green highlight).

The LINC complex resides in the nuclear envelope and connects the inner nucleoplasm with cytoskeletal components (Figure 1). The LINC complex consists of SUN proteins located in the inner nuclear membrane and KASH proteins located in the outer nuclear membrane (Stroud et al., 2014). SUN proteins span the inner nuclear membrane via a single transmembrane domain, with their N-terminal and C-terminal domains exposed to the nucleoplasm and the perinuclear space, respectively (Padmakumar et al., 2005; Crisp et al., 2006). The N-terminal domain of the SUN proteins binds nuclear lamins and chromatin-binding proteins in the nucleoplasm (Crisp et al., 2006; Haque et al., 2006; Chi et al., 2007; Wang et al., 2020) (Figure 2A). Lamin-associated proteins residing in the inner nuclear membrane, including emerin and TMEM201 (also known as NET5 or Samp1) also interact with SUN proteins and anchor the LINC complex in the nuclear envelope (Haque et al., 2010; Zhang et al., 2022). C-terminal SUN domains bind the KASH domain of KASH proteins in the perinuclear space. On the cytoplasmic side of the outer nuclear membrane, N-terminal regions of KASH proteins interact with all major cytoskeletal components including microtubules, actin, and intermediate filaments (Luxton and Starr, 2014).

SUN1 and SUN2 are expressed in most mammalian cells, with three additional SUN proteins expressed primarily in male germ cells (Malone et al., 1999; Shao et al., 1999; Hodzic et al., 2004; Xing et al., 2004; Padmakumar et al., 2005; Göb et al., 2010). Mammals express six KASH domain-containing proteins, including the four nesprins (Nuclear envelope spectrin repeat proteins), nesprins-1 to 4, as well as KASH-5, and lymphoid-restricted membrane protein (LRMP, also known as Jaw-1 or KASH-6) (Behrens et al., 1994; Zhang et al., 2001; Wilhelmsen et al., 2005; Roux et al., 2009; Morimoto et al., 2012; Horn et al., 2013b; Kim et al., 2015). Nesprin-1 and 2 are ubiquitously expressed and were originally believed to only associate with actin filaments via their N-terminal actin binding domains (Zhang et al., 2001; Zhang et al., 2002). However, further research showed that nesprin-1 and 2 also link to microtubules indirectly through motor proteins that bind both microtubules and nesprins (Zhang et al., 2009; Yu et al., 2011)**.** Nesprin-3 is also ubiquitously expressed, and it has a plectin-binding domain that binds to intermediate filaments via plectin instead of an actin-binding domain (Wilhelmsen et al., 2005). Balanced forces from microtubules and intermediate filaments in cardiomyocytes act through nesprin-1/2 and nesprin-3, respectively, to maintain nuclear morphology and regulate gene expression (Heffler et al., 2020). Nesprin-4 is mainly expressed in secretory epithelial cells (Horn et al., 2013b) and can interact with the motor protein kinesin to mediate microtubule binding (Roux et al., 2009). KASH-5 expression is limited to germline cells where it interacts with the dynein/dynactin complex associated with microtubules during meiosis (Morimoto et al., 2012). LRMP, found in the outer nuclear membrane and in the endoplasmic reticulum (ER), interacts with microtubules, although the binding mechanism is unclear (Kozono et al., 2018; Okumura et al., 2023).

The LINC complex has a key role in force mechanotransduction between the nucleus and the cytoskeleton, although how this is accomplished mechanistically is not well understood. A wide range of cellular functions are attributed to the LINC complex, from regulation of DNA repair (Lawrence et al., 2016), chromatin architecture (Ghosh et al., 2021) and chromosome movement during meiosis (Ding et al., 2007), to functions far from the nucleus such as cell-substrate adhesion (Chancellor et al., 2010). The LINC complex regulates aspects of cell migration and polarization via poorly understood mechanisms that likely involve its control of nuclear shape and positioning, and the nucleus-centrosome connection (Lombardi et al., 2011; Banerjee et al., 2014; Heffler et al., 2020; Nishino et al., 2023). Consistent with their pivotal role in cellular functions, mutations in genes encoding LINC complex components or interacting partners are associated with pathological conditions known as nuclear envelopathies (Janin et al., 2017). Examples include Hutchinson-Gilford progeria syndrome (HGPS), Emery-Dreifuss muscular dystrophy (EDMD), and dilated cardiomyopathy (see below for more detail).

## 3 SUN proteins: key integrators of LINC complex function

SUN1 and SUN2 interact molecularly with the nucleus via their N-terminal domains, and with the cytoskeleton indirectly via interactions of their C-terminal domains with KASH proteins. Thus, they are key integrators in the LINC complex, but the interplay between these two major SUN proteins, and how they interact with other binding partners, are incompletely understood. They are described as having both unique and overlapping functions.

### 3.1 SUN protein structure and function

SUN1 and SUN2 were first identified as homologues of the *C. elegans* proteins UNC-84A and UNC-84B, respectively (Malone et al., 1999). The SUN domain of SUN proteins is highly conserved throughout evolution (Fridkin et al., 2009). Human SUN1 and SUN2 share 46% overall amino acid sequence similarity that varies among domains, with the KASH-binding SUN domains having high conservation (82% similarity), while lamin-binding domains have low conservation (19.7% similarity) (Figure 2B). These sequence disparities and similarities likely account for the distinct vs. shared binding affinities of individual SUN proteins for lamins and nesprins, respectively. The precise mechanism by which the LINC complex links the cytoskeleton and the chromatin remains unclear. It is uncertain whether these interactions occur directly through the SUN proteins or indirectly via interactions with lamin-A or emerin, both of which can bind chromatin proteins (Lee et al., 2001; Gesson et al., 2016). SUN1 directly binds chromatin and DNA via its N-terminal domain in *Dictyostelium discoideum* (Xiong et al., 2008)*.* Although SUN1 directly binds DNA in lower eucaryotes, there is no evidence of direct binding between SUN proteins and DNA in mammalian cells. However, chromatin binding and immunoprecipitation assays revealed interaction of SUN1 with histone H2B in HeLa cells (Chi et al., 2007) and of SUN1 and SUN2 with DNA-dependent protein kinase complex in 293T cells (Lei et al., 2012). Both SUN1 and SUN2 interact with telomere-associated proteins such as MAJIN to tether telomeres to the nuclear envelope during meiosis (Schmitt et al., 2007; Wang et al., 2020; Chen et al., 2021). Both SUN1 and SUN2 interact with lamin-A and to a weaker degree with lamin-B1/C via their N-terminal domains (Crisp et al., 2006; Haque et al., 2006). However, SUN1 and SUN2 are differentially anchored to the nuclear lamina, with FRET (Fluorescence Resonance Energy Transfer) analysis revealing a stronger SUN1/lamin-A association than SUN2/lamin-A association (Östlund et al., 2009). Moreover, co-immunoprecipitation analysis in HeLa cells revealed that B-type lamins interact with SUN1 and not with SUN2 (Nishioka et al., 2016). Both SUN1 and SUN2 bind to type-A lamin-interacting proteins such as emerin (Haque et al., 2010). In addition, SUN1, and not SUN2, is closely associated with nuclear pore complexes and contributes to mRNA export in HeLa cells (Liu et al., 2007; Li et al., 2017). SUN1 association with nuclear pore complexes requires both SUN1 nucleoplasm and transmembrane domains (Liu et al., 2007).

The KASH-binding domains of human SUN1 and SUN2 are highly conserved, and both similar and distinct interactions between SUN and KASH proteins have been described. Both SUN1 and SUN2 bind all six mammalian nesprins (Padmakumar et al., 2005; Crisp et al., 2006; Ketema et al., 2007; Stewart-Hutchinson et al., 2008; Morimoto et al., 2012; Cruz et al., 2020; Gurusaran and Davies, 2021). Structural analysis showed that SUN1 and SUN2 form trimers in the nuclear membrane, with each trimer interacting with three KASH proteins (Sosa et al., 2012; Wang et al., 2012; Zhou et al., 2012; Jahed et al., 2021; McGillivary et al., 2023) (Figure 1). Early co-immunoprecipitation experiments suggested that SUN1 and SUN2 can bind each other and potentially form heterooligomers (Wang et al., 2006; Lu et al., 2008), while more recent crystallographic analysis suggested that SUN1 and SUN2 form homotrimers (Jahed et al., 2018a). Each SUN protein has two coiled-coil domains (CC1 and CC2), and these domains and the SUN domain extend into the perinuclear space (Nie et al., 2016; Gurusaran et al., 2023) (Figure 2A). The C-terminal SUN domain interacts with KASH proteins through a protruding structure in the SUN domain, called the KASH lid. In its monomeric state, the KASH lid of SUN proteins binds to CC2, which locks it in an autoinhibited conformation that prevents nesprin binding. Trimerization of the SUN proteins is likely mediated by CC1 and enables interaction between the KASH lid and nesprins (Nie et al., 2016). Although it is still unclear what triggers SUN protein trimerization, it may be dependent on calcium concentration (Jahed et al., 2018b; Majumder et al., 2022). Both SUN proteins form trimers and adopt an autoinhibited monomeric conformation. However, KASH lid inhibition of monomeric SUN1 is likely weaker than that of monomeric SUN2, suggesting that SUN1 may be more accessible for nesprin binding than SUN2 (Jahed et al., 2018a). These findings align with another study showing that SUN1 was consistently co-precipitated with both nesprin-1 and nesprin-2 while SUN2 was almost undetectable in nesprin immunoprecipitates, suggesting that SUN1 is more efficiently incorporated into the LINC complex (May and Carroll, 2018). However, plasmon surface resonance analysis did not reveal significant binding differences between SUN1 or SUN2 and the KASH domain of nesprin-2 (Östlund et al., 2009).

Although SUN1 and SUN2 have highly conserved KASH-binding domains, they differentially engage the cytoskeleton, suggesting that molecular differences in SUN-KASH interactions have consequences for cellular behaviors. SUN1 and SUN2 regulate nuclear positioning in fibroblasts via different mechanisms; SUN1/nesprin-2 complexes preferentially interact with microtubules to regulate forward nuclear movement, while SUN2/nesprin-2 complexes preferentially interact with the actin cytoskeleton to regulate rearward nuclear movement (Zhu et al., 2017). These findings align with another study showing that SUN2/nesprin-2 complexes assemble into Transmembrane Actin-associated Nuclear (TAN) lines that anchor nuclei to actin filaments during nuclear movement (Folker et al., 2011). Initial research indicated that LINC complex assembly into TAN lines was specific to SUN2/nesprin-2 complexes (Luxton and Starr, 2014), but subsequent work showed that other nuclear proteins such as nesprin-1, lamin-A/C, lamin-B1, and SUN1 are also concentrated in these lines (Versaevel et al., 2014; Hoffman et al., 2020; Smith et al., 2022). The mechanism by which SUN proteins discern specific nesprin-associated cytoskeletal components remains unclear, since there is no apparent specificity to nesprin/SUN binding. It is hypothesized that SUN proteins perceive force differences exerted on nesprins by different cytoskeletal components (Zhu et al., 2017). The distinction in SUN/cytoskeletal binding may be cell- or context-dependent, as SUN1 also regulates actin cytoskeleton organization (Ueda et al., 2022), while SUN2 is involved in astral microtubule organization during mitosis (Belaadi et al., 2022). Each SUN protein appears to regulate both the actin and microtubule cytoskeletal networks, yet it remains unclear whether any given effect is direct or indirect.

### 3.2 Function of the SUN proteins: findings from *in vivo* models

Early studies in mouse models demonstrated both distinct and redundant functions of SUN1 and SUN2 in developmental processes. Mice globally lacking *Sun1* are completely sterile due to severe impairment of telomere attachment to the nuclear envelope during meiosis and repressed gametogenesis-related gene expression (Ding et al., 2007; Chi et al., 2009). Similar to SUN1, SUN2 also localizes to telomere attachment sites and contributes to telomere tethering to the nuclear envelope (Schmitt et al., 2007). However, although SUN2 is present at attachment sites, it does not compensate for *Sun1* loss during meiosis (Link et al., 2014), suggesting distinct SUN-telomere interactions. *Sun1* loss in mice also disrupts nuclear localization in outer hair cells of the ear and leads to progressive hearing loss (Horn et al., 2013a). In contrast to mice globally lacking *Sun1*, mice globally lacking *Sun2* are fertile and have no apparent abnormalities in growth and development (Lei et al., 2009). However, a recent aging study revealed that *Sun2* mutant mice developed cardiac hypertrophy characterized by enlarged cardiomyocytes with altered sarcomere structure and adhesion. Despite these changes, no cardiac-related deaths were observed. Interestingly the global loss of *Sun2* in mice did not induce cardiac fibrosis. Instead, it led to the downregulation of TGFβ-associated profibrotic gene transcription in the left ventricle, attributed to elevated levels of MAN1, a negative regulator of the TGFβ signaling at the nuclear envelope (Stewart et al., 2019). The global loss of both *Sun1* and *Sun2* is lethal neonatally, indicating that SUN1 and SUN2 have overlapping functions developmentally. Redundant roles were found in regulating skeletal muscle nuclear anchorage (Lei et al., 2009) and coupling the centrosome and nucleus during neuronal migration in cerebral cortex neurogenesis (Zhang et al., 2009). Postnatally, decrease in cerebellar volume and impairment of motor coordination resulting from *Sun1* loss were exacerbated by the loss of one allele of *Sun2* (Wang et al., 2015). Embryonic fibroblasts isolated from mice globally lacking both *Sun1* and *Sun2* exhibited increased apoptosis and DNA damage due to compromised DNA damage response. This implies a redundant function of SUN1 and SUN2 in DNA repair, as no evident defects were observed in mice lacking either *Sun1* or *Sun2* individually (Lei et al., 2012).

### 3.3 LINC complex and SUN proteins in the vascular endothelium

The LINC complex is important for vascular development and homeostasis, and several recent studies address LINC complex functions in endothelial cell biology. Overall disruption of the LINC complex via dominant negative KASH (DN-KASH) over-expression *in vitro* revealed regulation of migration and responses to flow-mediated forces in Human Umbilical Vein Endothelial Cells (HUVEC) (Denis et al., 2021). LINC complex disruption through individual depletion of nesprin-1, 2 or 3 in endothelial cells revealed LINC complex involvement in endothelial cell behaviors. Depletion of nesprin-1 or nesprin-2 in HUVEC increased cell spreading and decreased endothelial cell network formation on Matrigel and collective endothelial cell migration (Chancellor et al., 2010; King et al., 2014). During collective cell migration, nesprin-1-depleted endothelial cells had impaired nuclear-centrosomal polarization at the leading edge and abnormal reorientation in response to cyclic strain that correlated with increased focal adhesion number. Traction force microscopy analysis showed increased traction forces in nesprin-1-depleted endothelial cells, leading to a model whereby disrupted nucleus-cytoskeleton connections downstream of nesprin-1 depletion led to reduced nuclear actomyosin forces and increased cytoskeletal tension on focal adhesions (Chancellor et al., 2010). Depletion of nesprin-1 and 2 in HUVEC demonstrated their necessity for shear stress-induced tight junction protein expression. Specifically, nesprin-1 and 2 co-depletion reduced the induction of ZO-1 (Zonula Occludens-1) and occludin protein expression by pulsatile shear stress (Yang et al., 2020). Nesprin-1 depletion in HUVEC rescued impaired barrier function resulting from *SUN1* depletion without showing obvious effects when depleted in the presence of SUN1, indicating a role for nesprin-1 in endothelial cell barrier function (Buglak et al., 2023). In rat aortic endothelial cells, both nesprin-2 and lamin-A protein expression decreased with exposure to low-shear stress (5 dynes/cm^2^) conditions, compared to arterial shear stress conditions (15 dynes/cm^2^), suggesting that these proteins respond to flow-mediated inputs. Lamin-A or nesprin-2 overexpression decreased cell proliferation and apoptosis under low-shear stress conditions (Han et al., 2015).

Dissecting the roles of nesprin-1 vs. nesprin-2 in endothelial cells has been challenging, as both proteins bind to SUN proteins and interact with both actin and microtubule networks. Nesprin-3 binds both SUN proteins (Ketema et al., 2007), but interacts with vimentin-containing intermediate filaments on the cytoplasmic side, leading to some clarity regarding its function in endothelial cells. In nesprin-3-depleted Human Aortic Endothelial Cells (HAEC), perinuclear vimentin loss is associated with impaired nucleus-MTOC (Microtubule-Organizing Center) connectivity, leading to nucleus-MTOC polarization defects under shear stress conditions. Moreover, nesprin-3 silencing induced endothelial cell hyper-elongation in the absence of stimuli (Morgan et al., 2011). Thus, nesprins are crucial for proper endothelial cell behaviors and for flow-mediated responses *in vitro*; however, the redundancy in binding and function, and the lack of *in vivo* vascular manipulations, have hindered explicit understanding of their unique roles in vascular function and detailed mechanistic understanding.

Focused research to decipher SUN protein function in endothelial cell biology has also been complicated. In non-endothelial cell types, SUN1 and SUN2 have both overlapping and unique functions in cell behaviors that are also involved in blood vessel formation and function, such as nuclear positioning and movement (Zhang et al., 2009), nucleus-centrosome connection (Chang et al., 2019; Lima et al., 2024), Golgi organization (Hieda et al., 2021), focal adhesion assembly (Ueda et al., 2022) and cell migration (Nishioka et al., 2016; Nishino et al., 2023). Studies of isolated nuclei improved our understanding of nucleus-cytoskeleton force transmission, revealing that force applied to nesprin-1 in HUVEC nuclei induces nuclear stiffening (Guilluy et al., 2014). Further work in fibroblast nuclei demonstrated that co-depletion of both *SUN1* and *SUN2* affected force transduction via nesprin-1, whereas individual depletion of either *SUN1* or *SUN2* did not show significant effects, indicating overlapping function. Studies on enucleated HUVEC and fibroblasts highlighted the role of the nucleus in maintaining cell contractility and traction forces that were dependent on substrate rigidity (Graham et al., 2018). Enucleated HUVEC and *SUN1*/*SUN2* co-depleted fibroblasts showed similar cellular phenotypes, with a decrease in cell contractility and traction forces. The co-depletion of *SUN1/SUN2* affected traction forces differently than nesprin-1 depletion (Chancellor et al., 2010), perhaps due to cell type or experimental differences. These findings indicate that SUN proteins regulate force transmission between the substrate and the nucleus via the cytoskeleton.

Force transmission between the substrate and nucleus induces chromatin deformation, resulting in changes in gene transcription. In CHO (Chinese Hamster Ovary) cells, localized surface force application propagated a signal from integrins to the nucleus via the actin cytoskeleton, leading to chromatin stretching and gene transcription (Tajik et al., 2016). The LINC complex contributes to this force transmission, as depletion of both *SUN1* and *SUN2* inhibited force-induced chromatin stretching. Disrupting the LINC complex in cardiomyocytes through the overexpression of a truncated nesprin-3 protein interfered with chromatin reorganization during cardiomyocyte maturation by decreasing the nuclear peripheral accumulation of trimethylated H3K9-marked chromatin (Seelbinder et al., 2021). During mitosis, *SUN1* depletion in HeLa cells decreased histone acetylation and delayed chromosome de-condensation (Chi et al., 2007). SUN2 modulates chromatin structure indirectly by interacting with lamin-A/C to maintain chromatin in a repressive state (Sun et al., 2018). Although SUN proteins regulate chromatin structure, their impact on gene transcription remains unclear. Whole genome expression profiling revealed few significant differentially expressed genes following *SUN1* depletion in HeLa cells or HUVEC (Li et al., 2017; Buglak et al., 2023). However, in HeLa cells, SUN1 regulates gene expression independently of its interaction with KASH proteins (May and Carroll, 2018). Moreover, disrupting the LINC complex in cultured fibroblasts using a SUN1 dominant-negative construct revealed its role in regulating gene transcription in response to substrate rigidity (Alam et al., 2016).

We recently showed that endothelial cell-specific deletion of *Sun1* led to impaired vascular expansion and endothelial barrier function in the murine postnatal retina, and that vascular sprouting was compromised in zebrafish lacking *sun1* (Buglak et al., 2023). Live imaging of 3D angiogenic sprouting showed that SUN1 negatively modulates sprout retractions, suggesting that depletion leads to increased contractility. The mechanism was revealed in HUVEC, where *SUN1* stabilized peripheral microtubules that in turn sequestered Rho GEF-H1 and negatively regulated Rho-mediated signaling. The effects of SUN1 depletion on endothelial junctions were rescued by co-depletion of either Rho GEF-H1 or nesprin-1. Taken together, the findings suggest that endothelial SUN1 functions as a decoy to negatively regulate nesprin-1-mediated LINC complex interactions that in turn regulate microtubule stability. Microtubule dynamics in turn regulate peripheral Rho signaling that modulates contractility, cell-cell junctions, and barrier function in endothelial cells. This model of nucleus-to-junctions molecular communication is consistent with other studies in non-endothelial cells; for example, SUN1 inhibited RhoA activation and focal adhesion assembly in HeLa cells by antagonizing SUN2-LINC complexes (Thakar et al., 2017). Moreover, SUN2 depletion rescued the Golgi dispersion of SUN1-depleted HeLa cells, suggesting that SUN1 suppresses SUN2/nesprin-2 complexes to maintain proper Golgi structure (Hieda et al., 2021).

The role of SUN2 is less investigated in endothelial cells, but several recent studies point to a required function in flow-mediated responses. SUN2/nesprin-1 interactions were increased in HUVEC exposed to pulsatile shear stress compared to oscillatory shear stress (Yang et al., 2020). It was hypothesized that the nuclear translocation of β-catenin is downstream of these interactions and regulates tight junction protein expression. TMEM201, an inner nuclear membrane-resident protein, interacts with SUN2 and lamin-A in endothelial cells and functions in the regulation of endothelial cell migration and angiogenic processes *in vivo* (Zhang et al., 2022).

## 4 The LINC complex and cardiovascular pathologies

Mutations in LINC complex components and their binding partners, including SUN1 and 2, nesprin-1 and 2, lamin-A and emerin, are associated with human disease. Hutchinson-Gilford progeria syndrome (HGPS), Emery-Dreifuss muscular dystrophy (EDMD), and dilated cardiomyopathy (DCM) are three diseases associated with mutations in components or interactors of the LINC complex that have cardiovascular pathology (Méjat and Misteli, 2010). The mutations affect vascular endothelial and smooth muscle cells in HGPS and primarily affect skeletal and cardiac muscle in EDMD and DCM, leading to cardiovascular disease.

### 4.1 LINC complex and Hutchinson-Gilford progeria syndrome (HGPS)

HGPS is a rare genetic disorder that causes premature aging, and children with HGPS have accelerated atherosclerosis, resulting in premature death in their early teens due to heart attack or stroke (Makous et al., 1962; Hennekam, 2006; Olive et al., 2010; Prakash et al., 2018). The disease results from an autosomal dominant mutation in the *LMNA* gene, leading to expression of a truncated and farnesylated form of lamin-A known as progerin (De Sandre-Giovannoli et al., 2003; Eriksson et al., 2003). Lamin-A is typically produced from a precursor protein called prelamin-A, which undergoes post-translational modifications, including farnesylation of the C-terminal region (Sinensky et al., 1994). Subsequently, the ZMPSTE24 (Zinc Metallopeptidase STE24) protease cleaves the C-terminal 15 amino acids, including the farnesyl group, resulting in the formation of mature lamin-A (Corrigan et al., 2005). Many HGPS patients carry a *de novo* mutation (c.1824C→T; p.G608G) in exon 11 of the *LMNA* gene. This mutation preserves the farnesylation site but activates a cryptic splice site, leading to the deletion of 150 nucleotides that includes the ZMPSTE24 proteolytic cleavage site in exon 11. Consequently, truncated and farnesylated progerin, without post-translational modification, accumulates at the nuclear envelope.

The cardiovascular deterioration observed in HGPS patients mirrors that of normal aging, although exposure to traditional risk factors is limited (Gordon et al., 2005; Merideth et al., 2008; Gerhard-Herman et al., 2012). Like aged individuals, HGPS patients develop atherosclerosis characterized by vascular smooth muscle cell (VSMC) loss in the media as well as vascular calcification and stiffening, leading to plaque erosion and rupture (Stehbens et al., 1999; Stehbens et al., 2001; Hamczyk and Andrés, 2019). Mouse models that recapitulate aspects of HGPS have been developed (Hamczyk et al., 2018a; Benedicto et al., 2021). Knock-in mouse models carrying a mutant *Lmna* allele deleted for exon 9 (*LmnaΔ9*) (Mounkes et al., 2003; Hernandez et al., 2010) or with a deletion of intron 10, intron 11 and the last 150 nucleotides of exon 11 (*Lmna* ^HG^) (Yang et al., 2005; Yang et al., 2006) result in expression of a truncated form of lamin-A. Mice globally expressing the human G608G mutation were generated via transgenesis (*G608G BAC*) (Varga et al., 2006) or via mutation of the endogenous locus (*Lmna* ^*G609G/G609G*^) (Osorio et al., 2011). The breeding of *Lmna* ^*G609G/G609G*^ mice to apolipoprotein E-deficient mice (*ApoE* ^−/−^) revealed progerin-induced acceleration of atherosclerosis (Hamczyk et al., 2018b). Two different *Zmpste24*-deficient mouse models (*Zmpste24* ^−/−^) have also been used to study HGPS (Bergo et al., 2002; Pendás et al., 2002).

Analysis of these models revealed that progerin expression affects both endothelial cells and vascular smooth muscle cells. While the effects of progerin expression on vascular smooth muscle cell properties are well documented, the effects of progerin on endothelial cell function, and the role of endothelial cells in HGPS development, is less understood. Mice with endothelial cell-specific expression of progerin had accelerated aging and a shortened life span associated with cardiac hypertrophy and fibrosis (Osmanagic-Myers et al., 2019; Sun et al., 2020), showing that endothelial cells contribute to the pathology of HGPS. Medial VSMC loss seen in some global HGPS mutants was not documented in endothelial cell-expressing progerin mice, suggesting that some cardiovascular impairment in HGPS is due to progerin expression in medial VSMC (Osmanagic-Myers et al., 2019). These findings align with another study showing that progerin expression in VSMCs induced arterial stiffness and inward remodeling, which were not observed in mice expressing progerin exclusively in endothelial cells (del Campo et al., 2019).

Ubiquitous progerin expression in mice leads to endothelial dysfunction. *Ex vivo* aortic wire myography demonstrated that *Lmna* ^*G609G/G609G*^ mice had impaired aortic contraction and endothelial-dependent relaxation (del Campo et al., 2020). However, restricting progerin expression to only VSMC or endothelial cells was not sufficient to induce impairment of endothelium-dependent relaxation, suggesting complex cell-cell interactions in the phenotype. Other *in vivo* studies showed that endothelial-specific progerin expression induced endothelial cell dysfunction characterized by cellular senescence, a pro-inflammatory profile, and downregulation of eNOS expression (Osmanagic-Myers et al., 2019; Sun et al., 2020; Manakanatas et al., 2022). Reduced eNOS activity and reduced nitric oxide levels were found in induced pluripotent stem cell (iPSC)-derived endothelial cells from HGPS patients, along with increased expression of pro-inflammatory molecules and reduced flow-induced gene expression (Atchison et al., 2020; Gete et al., 2021), suggesting that progerin perturbs endothelial flow-mediated responses and promotes inflammation. Progerin expression in endothelial cells also leads to reduced neovascularization following ischemia *in vivo* (Sun et al., 2020) and vascular network formation *in vitro* (Bonello-Palot et al., 2014; Gete et al., 2021).

Progerin-induced endothelial cell dysfunction in cultured cells has been studied using multiple approaches to induce progerin expression, including viral-mediated progerin expression, siRNA-mediated *ZMPSTE24* depletion, or treatment with Atazanavir, a protease inhibitor that results in accumulation of farnesylated prelamin-A. Progerin expression in cultured endothelial cells induced pro-inflammatory gene expression and increased oxidative stress leading to premature cellular senescence (Bonello-Palot et al., 2014; Bidault et al., 2020). Moreover, progerin-expressing endothelial cells displayed impaired shear stress responses. Progerin expression in HUVEC resulted in nuclear abnormalities and significant cell loss under laminar shear stress (Danielsson et al., 2022). Another study demonstrated decreased collective cell migration in progerin-expressing endothelial cells under laminar shear stress (Jiang and Ji, 2022). Thus, endothelial cell progerin expression induces dysfunction that likely contributes to cardiovascular alterations.

**FIGURE 3:**
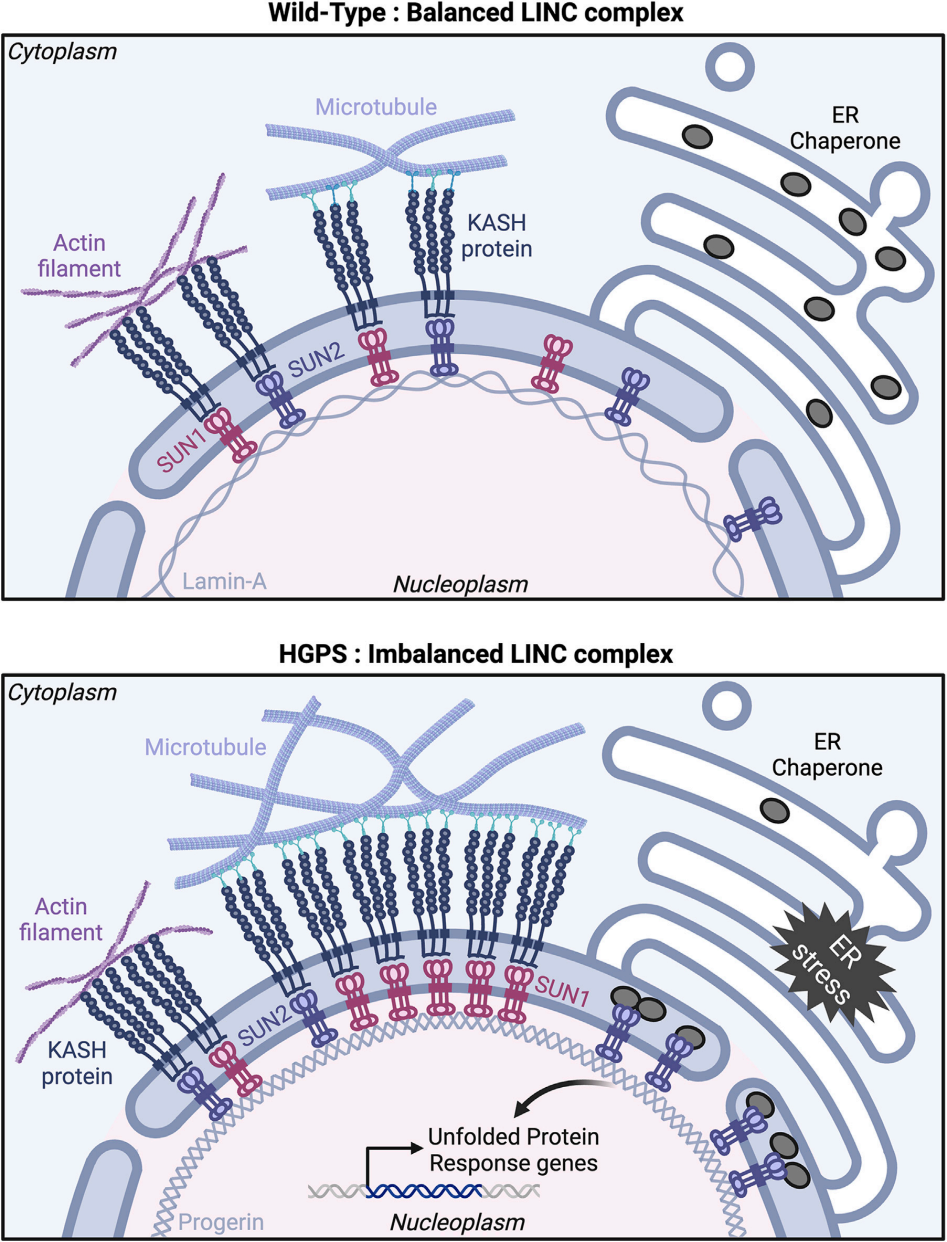
The nuclear LINC complex in health and disease. Model showing proposed interactions among LINC complex components in Wild-Type (top panel) and Hutchinson-Gilford progeria syndrome (HGPS) (bottom panel) cells. **Top**: In Wild-Type, SUN/KASH protein complexes engage similarly with actin filaments and microtubules, resulting in balanced interactions between the nucleus and the cytoskeleton. **Bottom**: In HGPS, progerin expression leads to SUN1 and SUN2 accumulation at the nuclear envelope, causing an imbalance in LINC complexes with a preference for interaction with microtubules over actin filaments. SUN2 also clusters at the nuclear envelope/ER interface, sequestering Endoplasmic Reticulum (ER) chaperone proteins and inducing an ER stress response. Created with BioRender.com.

Although the precise mechanisms by which *LMNA* mutations impact endothelial cell function in HGPS remain unclear, they likely involve lamin-A interactions with the SUN proteins that disrupt LINC complex-mediated nucleus-cytoskeletal connections. SUN1 expression is upregulated in progerin-expressing endothelial cells (Osmanagic-Myers et al., 2019), and in HGPS fibroblasts (Chen et al., 2012). Moreover, progerin farnesylation increased its binding affinity for SUN1, resulting in abnormal clustering of SUN1 and progerin in the endoplasmic reticulum (Chen et al., 2014). In this study, progerin expression reduced SUN1 motility in the nuclear envelope of HeLa cells, although no effect on SUN1 diffusional mobility was reported in HGPS patient-derived fibroblasts (Chang et al., 2019). *In vivo*, genetic loss of *Sun1* reduced disease severity and extended the lifespan of *LmnaΔ9* HGPS mice, indicating SUN1 involvement in HGPS pathology. Additionally, *SUN1* siRNA treatment rescued cellular senescence observed in fibroblasts from HGPS patients (Chen et al., 2012). Cell polarity defects of HGPS patient-derived fibroblasts in culture were rescued by siRNA-mediated depletion of *SUN1*, leading to a model whereby imbalanced LINC complexes downstream of *LMNA* defects resulted in impaired nucleus-cytoskeleton connections (Chang et al., 2019). In this model, increased levels of SUN1 compete with SUN2 for a limited pool of nesprin-2, thereby restricting the formation of SUN2/nesprin-2 complexes and changing the balance of LINC complex-mediated microtubule vs. actin cytoskeleton interactions (Figure 3).

SUN2 accumulates in progerin-expressing endothelial cells (Osmanagic-Myers et al., 2019) and in muscle cells from *Zmpste24* ^−/−^ mice (Yue et al., 2023), although how this contributes to HGPS pathology is not understood. In mesenchymal stromal cells from *Zmpste24* ^−/−^ mice, siRNA-mediated depletion of *Sun2* rescued the nuclear damage and cellular senescence induced by mechanical stress (Yue et al., 2023) suggesting that SUN2 mediates mechanotransduction. Progerin expression induces SUN2 clustering in the inner nuclear membrane in cultured fibroblasts. Moreover, SUN2 but not SUN1 depletion rescued an endoplasmic reticulum (ER) stress phenotype induced by progerin expression in fibroblasts. SUN2 clusters trigger ER stress inducing elevated UPR (Unfolded Protein Response) gene transcription downstream of progerin expression, leading to cell death. Interestingly, the UPR gene transcription induced by SUN2 clusters relies on the SUN2 C-terminal luminal domain rather than its nucleoplasmic and transmembrane domains (Vidak et al., 2023) (Figure 3).

### 4.2 LINC complex and Emery-Dreifuss muscular dystrophy (EDMD)

Patients with Emery-Dreifuss muscular dystrophy (EDMD) have muscle weakness and wasting that ultimately leads to premature death from cardiac abnormalities such as conduction defects and arrhythmia (Emery, 2000). Mutations in the *EMD* gene that encodes emerin, a LINC complex-associated protein, account for 60% of EDMD cases and are responsible for X-linked EDMD (Bione et al., 1994). Most *EMD* gene mutations are loss-of-function mutations resulting in the complete absence of emerin in the nuclear envelope (Koch and Holaska, 2014), while an autosomal dominant form of EDMD is associated with mutations in *LMNA* (Bonne et al., 1999). Screening of *SYNE1* (nesprin-1), *SYNE2* (nesprin-2), and *SUN1* and *SUN2* genes identified variants in patients with EDMD or related myopathies (Zhang et al., 2007; Meinke et al., 2014). Variants in LINC complex-encoding genes may explain the heterogeneity in disease severity, as EDMD patients with mutations in *SUN1* or *SUN2*, along with mutations in other genes like *EMD* or *LMNA*, had more severe symptoms compared to relatives without SUN gene mutations, although the *SUN* mutations alone were not linked to disease in other family members. Disruption of coupling between the nucleus and the cytoskeleton was highlighted by impaired centrosome reorientation in fibroblasts expressing SUN1 or SUN2 variant proteins (Meinke et al., 2014). Therefore, SUN1 and SUN2, by maintaining nuclear-cytoskeletal connections, regulate nuclear positioning and contribute to the pathogenicity of EDMD.

### 4.3 LINC complex and dilated cardiomyopathy (DCM)

Dilated cardiomyopathy (DCM) is defined by reduced systolic cardiac function resulting from enlargement and thinning of the left ventricular wall. DCM patients often develop heart failure, which can lead to sudden cardiac arrest (Hershberger et al., 2010). While the genetic basis of much DCM is unknown, mutations in the *TTN* gene which encodes the sarcomeric protein titin are associated with some DCM (Herman et al., 2012), and *LMNA* mutations represent the second most common genetic cause of DCM (Goidescu, 2013). Cardiomyocyte-specific deletion of *Lmna* in mice led to cardiac failure and decreased lifespan (Chai et al., 2021). The expression of the *LMNA* mutations found in DCM patients in human iPSC-derived cardiomyocytes disrupts peripheral chromatin organization, leading to the expression of non-myocyte lineage genes (Shah et al., 2021). Similar to the HGPS model, the global loss of *Sun1* rescued cardiac function and increased life span in these mice while the global loss of *Sun2* did not affect their longevity (Chai et al., 2021). Adenovirus Associated Virus 9 (AAV9) mediated injection of a dominant negative SUN1 miniprotein that disrupted binding between endogenous SUN and KASH domains also improved cardiac function and extended lifespan. These findings indicate the potential for future therapeutic treatments that reduce SUN1 accumulation.

## 5 Future directions

The LINC complex resides in the nuclear membrane, where it mediates nucleus-to-cytoplasm and cytoplasm-to-nucleus mechanotransduction and communication in ways that are only partially understood. Although we understand aspects of how the LINC complex is regulated and in turn how it regulates cellular behaviors, there are still gaps in our knowledge. Our understanding of the LINC complex involvement in vascular processes and contributions to cardiovascular disease is in the beginning stages. Cutting-edge assays to measure and manipulate molecular forces, along with advances in high-resolution cellular imaging and genetic manipulations and techniques, present exciting prospects for cardiovascular biologists investigating LINC complex interactions.

The LINC complex contributes to endothelial cell mechanotransduction, which is crucial for vascular function and impaired in diseases with compromised vascular function. However, the specific mechanisms whereby the LINC complex perceives forces and integrates with other cellular inputs remain unclear. Numerous studies highlight the importance of the nucleus in cytoskeletal regulation of cell behaviors such as migration and cell-cell adhesion, and the cytoskeleton in turn regulates nuclear movement within the cell. We predict that enhanced understanding of the underlying mechanisms will result from precise force measurements in different cellular locales. It will be especially important that these assays utilize physiologically relevant endothelial cell micro-environments to better understand effects in the cardiovascular system, as force generation and transduction depend on parameters such as substrate stiffness.

Another important property that may be elucidated via high-resolution imaging is the exact topology of the SUN protein complexes in the nuclear membrane. Do SUN1 and SUN2 form heterodimers or only homodimers? When and how do they interact with partners such as emerin? This knowledge is relevant for understanding the respective roles of SUN1 and SUN2 in endothelial cells and blood vessels, as published data overall suggests interactions that are sometimes complementary/redundant and sometimes antagonistic. The different interaction outcomes may be influenced by the relative location of SUN proteins in the nuclear membrane, and the clustering observed in HGPS and related diseases may alter the relationship between SUN1 and SUN2.

Finally, it will be exciting and important to test the role of the LINC complex and SUN proteins more rigorously *in vivo* to understand their effects on the cardiovascular system. Live imaging in zebrafish will help determine how the LINC complex regulates cell behaviors dynamically *in vivo*. Selective genetic manipulations in endothelial cells or other vascular cells in mice will help to understand how each cell type contributes to premature aging and cardiovascular disease, and profiling of vascular cells from animal models will also help define roles. We anticipate that addition of other -omics (such as ATAC-seq, proteomics, etc.) will complement RNA profiling, and spatial -omics will add a further dimension of understanding to the pathologies resulting from nuclear and LINC complex perturbations. Once there is a more unifying concept of how the LINC complex participates in endothelial cell behaviors and blood vessel function, rational design of therapeutics to mitigate the effects of disease and perhaps normal aging will be closer to reality.
